# Friend or Foe? Early Social Evaluation of Human Interactions

**DOI:** 10.1371/journal.pone.0088612

**Published:** 2014-02-19

**Authors:** Marine Buon, Pierre Jacob, Sylvie Margules, Isabelle Brunet, Michel Dutat, Dominique Cabrol, Emmanuel Dupoux

**Affiliations:** 1 Laboratoire de Sciences Cognitives et Psycholinguistique, Ecole Des Hautes Etudes en Sciences Sociales, Département d’Etudes Cognitives-Ecole Normale Supérieure, Centre National de la Recherche Scientifique, Paris, France; 2 Maternité Port-Royal, Faculté de Médecine Port Royal-Cochin, APHP, Université René Descartes, Paris, France; 3 Institut Jean Nicod, Ecole Des Hautes Etudes en Sciences Sociales, Département d’Etudes Cognitives-Ecole Normale Supérieure, Centre National de la Recherche Scientifique, Paris, France; Boston College, United States of America

## Abstract

We report evidence that 29-month-old toddlers and 10-month-old preverbal infants discriminate between two agents: a pro-social agent, who performs a positive (comforting) action on a human patient and a negative (harmful) action on an inanimate object, and an anti-social agent, who does the converse. The evidence shows that they prefer the former to the latter even though the agents perform the same bodily movements. Given that humans can cause physical harm to their conspecifics, we discuss this finding in light of the likely adaptive value of the ability to detect harmful human agents.

## Introduction

Humans seem unique in their ability to help, cooperate and communicate with their conspecifics [1], [2], and also to harm, defect and deceive them [3], [4]. Therefore, the ability to discriminate potentially benevolent from malevolent agents would seem important for survival, not only in adulthood but also in early childhood or even infancy. Congruent with this view, recent developmental studies indicate that infants do not only represent agents’ actions, goals and intentions [5]–[7], but also evaluate them: using non-verbal dependent measures, several studies have showed that young toddlers and even preverbal infants are able to *evaluate* some actions as either positive or negative and express *social preferences* towards agents as a function of the valence of their actions.

In a seminal study, Premack and Premack [8] showed 52-week-old infants interactions between pairs of 2D balls on a computer screen. In the habituation phase of the experiment, infants saw one ball performing either a negative action towards another ball (hitting or preventing the ball to achieve its goal) or a positive action (caressing or helping). Infants habituated to a positive action, but not to a negative action, showed a dishabituation response (as measured by looking times), when presented with a novel instance of a negative action (hitting). This suggests that 52-week-old infants are able to categorize actions along their positive or negative valence across differences in the low-level kinematic characteristics of the actions.

More recent studies went a step further by exploring whether infants are able to socially *evaluate* an agent as a function of his/her performed action. Hamlin, Wynn and Bloom [9] showed that 6- and 10-month-old preverbal infants prefer an agent whose action is congruent rather than incongruent with the goal of another agent climbing towards the top of a hill (see also [10], [11] for results with younger infants and other social scenarios). Nineteen-month-olds [12] and even 16-month-olds [13], [14] have also been shown to be sensitive to the fair/unfair allocation of resources among distinct individuals. Vaish, Carpenter & Tomasello [15] showed that young preschoolers are more prone to help an agent who did not intend to destroy another’s property than an agent who intended to perform this action, whatever the consequences of their actions (see [16] for similar results regarding physical harm; see also [17], [18] for the distinction between various benevolent/malevolent actions whatever the consequences).

Although infants show clear evidence of being able to socially evaluate the agents of some malevolent/benevolent actions, it remains to be established whether this ability stems from a generic capacity to evaluate an agent as a function of the *valence* of his/her action, as suggested by Premack and Premack [8], or whether it rests on a collection of domain-specific social evaluation systems [19]. It is possible that some pairs of benevolent/malevolent actions are more primitive or essential for survival than others and that agents who perform them are therefore evaluated more robustly and earlier by young infants than others. For example, the survival of young infants might depend more upon the contrast between harming and comforting than upon the contrast between fair and unfair allocations of resources. Even if there is a single action evaluation system in infants, it is clear that no simple set of perceptual cues can characterize the contrast between malevolent and benevolent actions in general. For example, the detection of a contrast between harming and comforting relies on the ability to perceive qualitative changes in individuals’ *emotional* or *physical* states. The detection of a contrast between helping and hindering actions requires the ability to understand an agent’s *goal* and whether this goal has been achieved or not. The detection of unfair distributions depends on the joint abilities to track the number and quality of recipients, the fraction of the commodity allocated to them, and some standard for assessing what counts as a *fair* distribution. The detection of a violation of a property right (the destruction of another’s property, for example) requires that infants have some understanding of the concept of *ownership* (or at least attachment) of an agent towards some inanimate objects, and that they keep track of them in third-party exchanges. Given that these situations implicate cognitive mechanisms of different complexity, it is unlikely that the corresponding evaluative capacities all emerge at the same time in human infants. It is therefore of considerable theoretical interest to establish separately, in each of these domains, the emergence of evaluative capacities in young infants.

In this paper, our aim is to investigate whether the ability to distinguish and socially respond to the harm/comfort contrast is achieved by the age of 10 months. Surprisingly, this contrast has, so far, not been studied independently. Smetana [20], Leslie and collaborators [17] et al. (2006), Weisberg & Leslie [18] and Vaish and collaborators [15] have studied preschoolers’ responses to situations of harm but they were mixed with violations of property rights. Nelson [16], Zelazo, Helwig & Lau [21] and others studied situations of harm independently, but they did not explore whether toddlers or even infants would socially evaluate benevolent/malevolent agents. Premack and Premack [8] showed that 52-week-old infants seem able to generalize from a harmful/comforting contrast to a helpful/hindering one, but they did not test whether infants are able to evaluate the agent of these actions. Hamlin et al. [9] showed that 10-month-old infants prefer a pro-social to an anti-social agent, but only for the hindering/helping contrast. Therefore, it is plausible that 10-month-old infants should be able to discriminate a harmful from a comforting agent, but it has not been tested yet whether they prefer the latter over the former.

One of the difficulties in studying a pure benevolent/malevolent contrast, and in particular a contrast like harm/comfort, is to construct a situation where infants’ or toddlers’ reactions depend on their conceptual understanding of the agents’ action or intentions, and not on the mere presence of superficial cues of positive or negative valence. For example, in Vaish’s study [15], the malevolent agent performs an anti-social action: he intentionally destroys the property of another character. In addition, he emits “mildly aggressive vocalizations”. As infants are able to discriminate different emotional expressions by an early age [22], [23] express preferences for positive emotions (happiness) over negative ones (sadness and anger) [24], the fact that the agent who intends to perform a bad action emits “mildly aggressive vocalizations” makes it difficult to know which aspect of the situation the toddler is reacting to (the superficial negative cues vs. the negative valence of the act of destruction). Similarly, in Hamlin’s experiment [9], after the puppet has been helped, it jumps up and down till the end of the sequence; this could be interpreted as a state of excitement. By contrast, after the puppet has been hindered, it rolls end-over-end down the hill and then remains immobile till the end of the sequence; this could be interpreted as a depressed state. The differences in the puppets’ motions in the final part of the sequences may reflect differences in underlying emotional or physical states, and could partially contribute to infants’ evaluations, irrespective of whether they understand helping and hindering at a conceptual level.

The purpose of our paper is to investigate the preferences of preverbal human infants when confronted with a contrast involving only harming and comforting. In order to enhance the ecological validity of cues available to infants, we embodied this contrast in movie clips using human agents performing simple actions (threatening and pushing to the ground, raising and comforting), rather than animated geometric shapes. As indicated above, the contrast between harming and comforting is particularly difficult to study, because it inherently incorporates a change in the victim’s emotional state, which is confounded with the anti-social versus pro-social nature of the action. In order to mitigate this confound, we equated the average emotional state of the human victims across all situations: in the harm condition, the victim is first happy, then sad; in the comfort condition, she is first sad, then happy. Thus, the absolute amount of positive/negative cues is matched, and the only possible cue relevant to evaluating the victim’s emotional state is in the *change of state* associated to the agent’s action. We then equated the actions and expressions of the anti-social and pro-social agents by using a cross-over, internal control design, in which the two agents each performed a pair of actions, one positive, one negative. Critically, the ‘pro-social’ agent directed his positive action towards a human “patient” and his negative action towards a non-human “patient” (an inanimate object), while the ‘anti-social’ agent did the opposite. In this way, the two agents exhibited the same overall amount of positive and negative emotions and the only way to distinguish them is to track the agent’s actions in conjunction with the human versus non-human status of the patient.

To examine whether toddlers and infants distinguish and socially respond to the harm/comfort contrast, we conducted two experiments. In Experiment 1, we tested 29-month-olds using verbal questionnaires designed to explore toddlers’ absolute evaluations of each agent taken individually as well as their preference toward one agent over another. In experiment 2, we tested the preferences of preverbal 10-month-olds for one agent over another using a non-verbal toy choice task previously used by Kinzler, Dupoux and Spelke [25].

## Experiment 1

In this experiment, 29-month-old toddlers saw short films involving two distinct adult agents: the anti-social agent and the pro-social agent. Each of these agents performed a pair of actions: one directed towards a human patient and the other towards an inanimate object. The anti-social actor threatened and pushed a little girl, and he caressed a backpack resting on a stool. Conversely, the pro-social agent comforted the little girl, and he threatened and pushed the backpack. The agents were two adult males and the human patient was a female child. The inanimate object was chosen so as to match the size of the human patient. In both pairs of positive and negative actions, we took care of matching both the bodily movements and emotional displays of agents of both types. Therefore, the only factor distinguishing the pro-social from the anti-social agents was how their two actions (positive vs. negative) were paired with the two targets (human patient vs. inanimate object) and the targets’ subsequent reactions (emotional changes for the human patient, and no visible changes for the inanimate object). In this experiment, the dependent variable was the response to verbal questionnaires administered through a puppet. As toddlers of this age tend to give inconsistent responses to the same questions, we used a total of 9 questions some of them regarding each agent individually and the others allowing the child to compare the two agents. We then combined the responses into absolute valence indexes (composed of the responses given by toddlers to the individual questions) and a relative valence index (composed of the responses given by toddlers to the comparative questions).

### Method

#### Stimuli

We designed four action scripts representing all possible combinations of the two action-types (harm/comfort) and two patients (human patients/inanimate object). Each action script had the same temporal structure that involved three phases: (i) the target is alone (8 sec), (ii) the agent enters the scene and interacts with the patient (14 sec),(iii) the patient is alone again (8 sec). In the human-harming action script, (i) the human agent is dancing and smiling while displaying other happy body language. (ii) The agent walks in, threatens the human patient while walking towards her, pushes her down to the floor, simulates kicking her, and leaves. (iii) The human patient is then lying on the ground and displays distress cues (crying). In the human-comforting action script, (i) the human patient is lying on the floor, displaying distress cues (crying). (ii) The agent walks in, raises the target up, comforts her (caressing her), smiles, and leaves. (iii) The human patient is executing a little dance with happy face and positive body language. Note that stage (i) of the human harming action script is the same as stage (iii) of the human comforting action and vice-versa. The object-harming and object-comforting action scripts are exactly the same as their counterparts with a human patient, with identical timing, action and emotions of the agent: he walks in, threatens while walking towards the inanimate object, pushes it down to the floor, simulates kicking it, and leaves vs. he walks in, raises the inanimate object up, comforts it - caressing it -, smiles, and leaves. Therefore, the only difference is that the human patient has been replaced by an inanimate object. The object, of course, does not display any emotions; it is in one of two states: either standing upright (the equivalent of the girl dancing) or lying on the ground (the equivalent of the girl lying and crying).

Each of the four action scripts was cast twice, using two different male adults as actors (actors A and B), resulting in eight 30-seconds movie clips. In all of the movie clips, the human patient was a female 12-year-old actor and the inanimate object was a backpack resting on a stool, whose size approximately matched the size of the human patient. The movie clips were silent, and were cast in a studio with a uniform blue background.

The movie clips were then mounted into sequences of two consecutive actions separated by a blank screen of two seconds. These sequences involved the same actor but different patients (human patient or inanimate object) and actions (harming or comforting). For instance, in an ‘anti-social’ sequence, actor A harms the little girl and then comforts the backpack. In a ‘pro-social’ sequence, actor B hits the object and then comforts the little girl. Eight such sequences were generated by crossing the two actors (A and B), the two roles (pro-social and anti-social), and two orders of presentation of actions (harming first or comforting first). Note that movies are available upon request to the main authors.

#### Procedure

Before the experiments, the toddlers were familiarized to the puppet character (animated by the experimenter) that will be used subsequently. The puppet played with them and asked them to name and point to various animals in a picture book (what is this animal? Where is the elephant? etc.). The toddlers were then placed two meters away from of a 2×1.5 m projection screen, in front of a table, on their parent’s lap. The parents were blindfolded during the entire experiment. The experimenter was seated on the ground facing away from the screen and was, therefore, blind to the movies being played.

The experiment consisted of three parts. In the first part, the toddlers were presented with one of the eight sequences (involving, for instance, actor A in the anti-social role, with the harming action first) played twice, with a 2-second blank screen interval between each repetition. This was followed by a photograph of the actor facing the camera, at which point the toddler was introduced to an experimenter blind to the film, who used the puppet to interact verbally with him/her and asked social/moral evaluation questions regarding the actor appearing in the photograph: Do you like him? Is he a good guy or a bad guy? Is he nice looking or ugly? Is he scary or nice? Do you want to play with him or not?

In the second part, toddlers were presented twice with a sequence involving the other actor playing the opposite role (here, actor B in the pro-social role, with the harming action first). As in the first part, the sequence was followed by a presentation of actor B’s photograph, and the same questions were used again.

In the third part, the two sequences with actor A and actor B were shown once and the two actors appeared side by side on the screen. When faced with the two actors, the child was asked one question for each agent “Is he a good guy or a bad guy?” while the puppet pointed first to the actor on the left, then to the actor on the right. Then, they were asked three questions using a contrastive construction: “which one is the nice guy?”, “which one is the bad guy?”, “which one would you like to play with?” For the contrastive questions, toddlers were requested to point to one of the two sides of the screen. In total, the children were therefore asked 6 individual questions for each of the two actors, plus 3 contrastive questions.

If the child failed to answer a question after 10 seconds of silence, the question was repeated. After 10 more seconds of silence, the question was considered as unanswered and thus as a missing data point in the analysis (average: 3.62 questions unanswered per toddler, SE: 0.68). If the child appeared to become agitated, or refused to answer any more, subsequent questions were skipped (average 1.23 questions skipped, SE: 0.41), and the next video sequences were played. On average, toddlers thus missed 4.85 questions (SE = 0.84). Note that we found no effect of the questionnaire (individual vs. contrastive), of the questions asked or of the counterbalanced factors on none of this factor). For a full description of the data obtained in experiment 1, see Table S1.

The choice of the actor playing the pro-social agent (actor A vs. actor B), the order of presentation of the pro-social agent (first vs. second) and the order of presentation of the action-type (harming vs. comforting) were counterbalanced across 8 groups of toddlers. Toddlers’ responses were video, tape-recorded and blindly scored by two independent coders (Cronbach’s alpha = .94). We used the average between the two coders ‘scores for subsequent analyses.

#### Participants and ethical issues

We tested forty-six 29-month-old toddlers (age range = 28 to 32; 23 males, 23 females) recruited in Paris through mailing and telephone calls. Upon recruitment on the phone, the parents were informed about the aims of the study and about the methodology. When parents arrived, these elements were explained again. The parents were then brought to the experimental room without the toddlers and shown clips of the 4 action scripts (with the same agents and same order for all parents). They were then asked whether they thought these clips were appropriate to their toddlers, and if so, were given the informed consent form to sign, and the experiment proceeded. During the experiment, the parent was blindfolded and given the option of stopping the experiment at any point. This study was approved by the Cochin-Tarnier Hospital Ethical Committee (Comité de protection des personnes “Ile-de-France III”, decision A01142-51).

### Results and Discussion

A total of eighteen toddlers could not be analyzed due to a technical failure in sound recording (N = 15), a complete absence of coherent or understandable responses (N = 2) and parental intervention during the questionnaire (N = 1). The responses of the remaining twenty-eight toddlers (14 males and 14 females) were analyzed.

Because this experiment was designed to analyze toddlers’ evaluation of each agent separately as well as their preference for one agent over another, we analyzed the responses obtained from the individual questionnaires and those from the comparative questionnaire separately.

For individual questionnaires, for each agent evaluated, we computed an Absolute Valence Index (AVI): each child’s response *in favor* of the agent (“yes, I like him”, “He’s nice”, “I want to play with him”, etc.) was coded as +1 while each response in disfavor of the agent (“no, I do not like him”, “He is mean”, etc.) was coded as −1. A score of 0 was assigned if the child gave no response or a response that was too ambiguous to code one way or the other. Then, we computed an aggregate Absolute Valence Index by averaging the code of the responses to each question.

For the contrastive questionnaire, we computed a Relative Valence Index (RVI) based on toddlers’ responses to comparative questions: each child’s response was coded as +1 if the child responded in favor of the pro-social agent or in disfavor of the anti-social agent, −1 if he or she gave the opposite responses. A score of 0 was assigned if the child gave no response or a response too ambiguous to code one way or the other. Here again, we computed an aggregate Relative Valence Index by averaging the code of the response to each question. The RVI was between −1 and +1, a positive value indicating that the “pro-social agent” was globally evaluated more positively than the “anti-social agent”, and vice-versa for a negative RVI.

**Figure 1:**
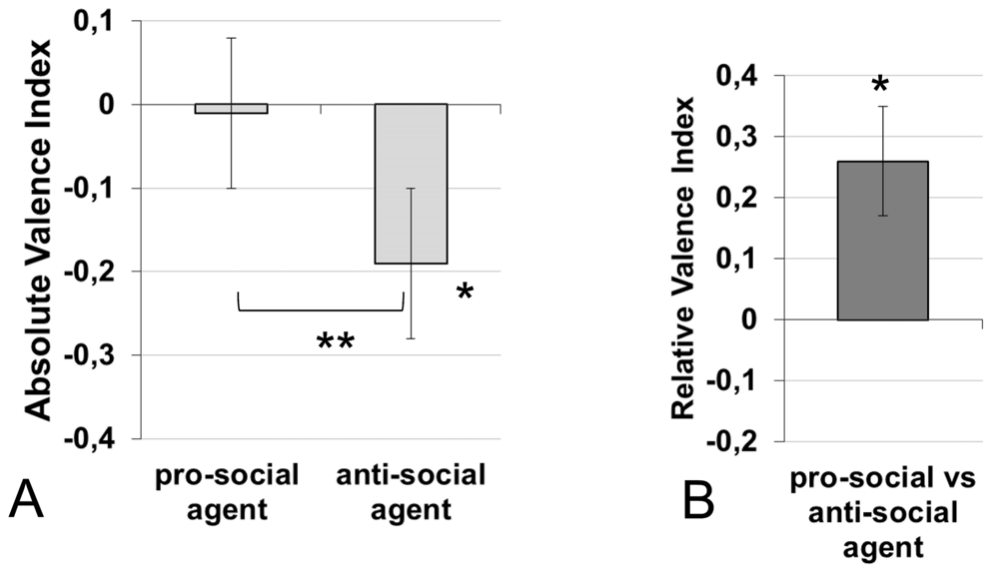
Average indexes for 29 months-olds. **A.** Average Absolute Valence Index for 29-months-old toddlers computed over the responses to the individual questions regarding the pro-social agent and the anti-social agent separately. *p<.05 (between subjects),**p<.02 (within subjects). A positive score indicates a positive verbal statement toward the agent and vice versa for negative scores. The error bars correspond to one between-subject standard error above and below the mean. **B.** Average Relative Valence Index computed over the responses to the contrastive questions. A positive score indicates a more positive assessment of pro-social than the anti-social agent, vice-versa for negative scores. The error bars correspond to one between-subject standard error above and below the mean. *p<.05.

The average AVI for each agent (pro-social vs. anti-social) across toddlers is shown in Figure 1.A. To start, we were interested to know if, irrespective of counterbalancing effects, toddlers found the pro-social agent to be positive and the anti-social agent to be more negative. To this end, we constructed a separate linear model for each agent type using the AVI as the dependent variable. Included in the model were the three counterbalancing factors (actor, order of actions and order of patient) and the resultant intercept was tested against zero. There were no effects of counterbalancing factor on either the pro-social or anti-social agents Furthermore, we found that the AVI obtained for the pro-social agent was slightly negative but not significantly different from zero (AVI = −0.01, SE = 0.09, F(1,27) <1, p>0.1, ηp^2^ = 0.001) whereas, the AVI obtained for the anti-social agent was negative and significantly below zero (AVI = −0.19, SE = 0.09, F(1,27) = 6.29, p<.05, ηp^2^ = .34).

We then ran a repeated-measures ANOVA to examine the difference between pro-social and anti-social AVI. This analysis indicated that the AVI differed significantly as a function of the agent, (F(1,12) = 10.71, p<.02, ηp^2^ = .47) such that the anti-social agent is evaluated more negatively than the pro-social one. No effects of counterbalancing factor or of participant sex were found in these analyses.

The average RVI across toddlers is shown in Figure 1.B and was analyzed using a linear model with the three counterbalancing factors (actor, order of actions, and order of patient) as between subject factors. As above, the intercept was tested against zero. This analysis revealed an intercept significantly above zero (RVI = 0.26, SE = 0.09, F(1,27) = 5.14, p<.05, ηp^2^ = .25) showing that toddlers evaluated the pro-social agent significantly more positively than the anti-social agent. Again, there were no effects of the counterbalancing factors, participant sex or any interactions between these factors (p>0.1).

Sixteen toddlers occasionally produced short comments during the movie clips (30 comments in total), which we analyzed by taking into account the valence of the comments (neutral, positive or negative) and the action script concerned (human harming, object harming, human comforting or object comforting). All comments which were a description of the action (e.g. “he pushes the girl!”, “the girl falls”) were considered as neutral, those containing positive words (e.g. “he’s nice!”) as positive comments and those containing negative words (“bad !”, “he’s not nice”) as negative comments. If a toddler repeated the same comment in a same sequence (for example: “he’s bad!”) twice, we coded it as a single comment.

A four by three contingency table was constructed (see Table 1), by tabulating the three types of comments across the four types of action scripts. There was a significant effect of comment type (X*^2^* (2) = 6.2, p<.05), reflecting the fact that toddlers gave mostly negative comments, and very little positive comments. There was a significant effect of action scripts (X*^2^* (3) = 17.2, p<.001), reflecting the fact that the human harming sequence generated the most comments. Finally, there was an interaction between these two factors (X*^2^* (6) = 13.1, p<.05), reflecting the fact that by far the most frequent comments were negative comments produced during the human harming sequences. This result indicates overall a greater sensitivity to the negative act performed towards the little girl.

**Table 1:** Number of positive, neutral or negative comments during the four types of action scripts.

	Action script			
	Human		Object	
Comments	Harming	Comforting	Harming	Comforting
Positivecomments	0	2	1	1
Neutralcomments	5	2	4	0
Negativecomments	12	1	1	1
Total	17	5	6	2

## Experiment 2

### Method

**Figure 2:**
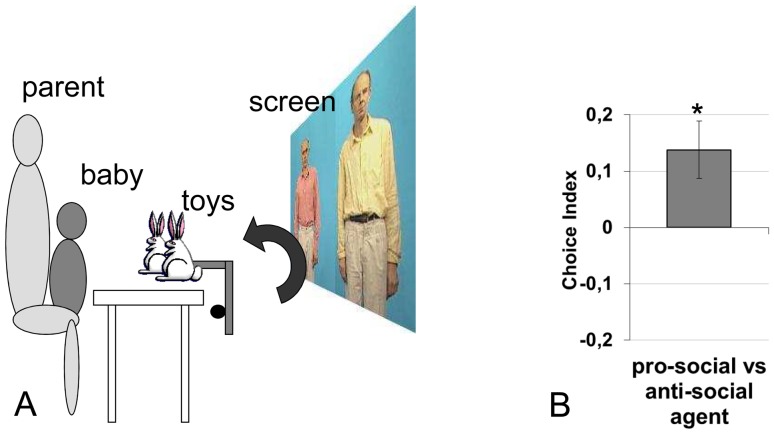
Set up and average preference index for 10-month-olds. A Setup of the experiment for the 10-month-olds. Infants are seated in their parent’s lap (who are blindfolded) during the experiment. After the presentation of the sequences described in Figure 1, infants see a novel sequence, where the two actors appear on stage, stare at the camera, play with two identical toys, and present them to the infants. Simultaneously, two real toys appear on the table through a mechanical device. The infants’ first attempt to reach one of the toys is coded by two independent scorers. Infants are tested 4 times with different toys, and the actors switching places on the screen. B. Average preference index for 10-month-olds computed over the 4 response trials. A positive score indicates a preference towards the toy of the pro-social agent, a negative score, a preference towards the anti-social agent. The error bars correspond to one between-subject standard error above and below the mean. *p<.05.

For the 10-month-old infants, we used the same design and stimuli as in Experiment 1, but the movies were recast in order to eliminate the simulated kicking (the negative act was reduced to pushing). Infants were presented with the same set of actions as before, but the questionnaires were removed. Instead, at the end of the entire set of movie clips, the two agents were shown entering the stage from each side, standing still facing the camera, playing with identical toys (teddy bears), and bending towards the camera as if both were simultaneously giving the toy to the infant. Through a mechanical apparatus (see Figure 2.A), the two identical toys then appeared on the table in front of the infant while it disappeared from the agents’ hands. Afterwards, the two agents appeared standing still with no toy, looking at the infant. This procedure was repeated four times (with four different teddy bears), with the agents swapping sides from trial to trial. The infants were videotaped and their initial attempts to reach for one of the toys were coded by two independent blind coders.

#### Ethical issues

The same procedure as in Experiment 1 was used, and was covered by the same decision from the Cochin Ethical Committee.

#### Participants

We tested fifty-four 10-month-old infants (age range = 9–10 months; 31 males, 23 females) recruited in Paris through mailing and telephone calls. The parents were fully informed about the protocol and were shown the sequences as in Experiment 1. The babies were seated in the lap of a parent, who was blindfolded during the entire experiment. Among the 10-month-old infants, 4 did not complete the presentation (unrest, technical failure), and 3 did not run the test session, leaving 47 infants to analyze (24 males, 23 females).

### Results and discussion

Several infants’ first attempts at reaching did not succeed either because the infant did not complete his or her gesture, or because they failed to remove the toy from the presentation arm. After an uncompleted reaching attempt, infants tended to reach towards the other object, and even subsequently to alternate between the two objects. Given such behavioral variability, we decided to code the first reaching attempt, towards one of the objects, whether or not the reaching was successful. Coders were instructed to use movements of one or both arms, as well as the orientation of body and head in order to determine the first reaching attempt. It was coded as 1, if directed towards the pro-social agent, and −1, if directed towards the antisocial agent, respectively. No obvious intended action towards one or the other toy after more than one minute was coded as zero The reliability between the two coders was.85 using Cronbach’s alpha, and.80 using Cohen’s Kappa (see Table S2 for row results). In order to analyze these responses statistically, the scores for the four trials (involving different toys) were averaged across the two coders to produce an average Choice Index (CI) for each infant (between −1 and +1): 57% of the infants had a positive Choice Index, 21% a negative one, and 21% were at zero (see Figure 2.B). An ANOVA of the Choice Index was run, declaring actor, order of the actions, and order of the patients as counterbalancing factors.

The intercept of the Choice Index was significantly above zero (index = 0.138; SE = 0.051, F(1,37) = 7.9, p<.008, ηp^2^ = .18) indicating that infants reached significantly more towards the object of the pro-social agent than the object of the anti-social agent. There was a marginal preference for one of the actors, (F(1,37) = 3.9, p = .06, ηp^2^ = .10) and no other counterbalancing factor introduced a significant effect or interaction (p>.1). A separate analysis showed no effect of the sex of the infant on the Choice Index (F<1, p>.1).

In order to assess the robustness of the choice index as a measure of preference, we ran separate analyses for each coder separately. We found a significantly positive intercept for both of them (index = 0.165, SE = 0.056, F(1,37) = 8.9, p<.005, ηp^2^ = 0.18 and index = 0.112, SE = 0.053: F (1, 37) = 4.7, p<.035, ηp^2^ = .10, respectively). The Choice Index as a dependent variable was obtained by averaging the results of 4 binary forced choices. As such, it may not respect the normality assumptions for an ANOVA. To check for this possibility, we ran a mixed model logistic regression on the probability of choosing the pro-social agent as opposed to the anti-social agent (1 for a pro-social choice, 0 for an anti-social choice and 0.5 for an indeterminate choice) on each of the four trials separately (the associated probabilities were averaged across the two coders). The logistic model was run with actor, order of the actions, order of the patients and trial number as regressors. We still found a significant intercept (Z = 2.06, p<.04), while no other factor reached significance.

## General Discussion

We investigated infants’ responses to the contrast between an anti-social agent and a pro-social agent: while the former pushed down a human patient and comforted an inanimate object, the latter comforted the human patient and pushed the inanimate object. The overall amount of aggressive/threatening cues and comforting/smiling cues displayed by both the pro-social and the anti-social agents were constant, as were the emotional expressions of the human patient. Therefore, the only difference between the actors was the recipient of their respective positive and negative actions. We found that the 10-month-olds chose more often the toy from the pro-social rather than the anti-social agent, while the verbal preferences of the 29-month-olds favored the pro-social agent compared to the anti-social agent.

### Low-level Explanation

The preference of toddlers and infants for the pro-social agent cannot be explained by intrinsic features of one actor over the other, as the actors’ roles are counterbalanced across subjects, and their overall movements and emotional displays are equated. Note that as pointed by an anonymous reviewer, one may wonder whether our actors displayed exactly the same emotional expressions irrespective of whether their actions were directed toward a little girl or a backpack. The response is that we did ensure that our actors’ display of positive and negative emotional expressions were the same whether their target was the little girl or the backpack (see Experiment S1).

Our results can neither be caused by mere associations between one of the agents and the human patient’s display of emotional cues, as both agents spent the same amount of time with the object and the human patient. Furthermore, the overall amount of positive and negative affects displayed by the human patient was equated across the pro-social and the anti-social agents’ actions. Finally, recency effects can also be discarded because the order between both agents and actions were counterbalanced across our participants.

Thus, what our experiment shows is that toddlers and infants prefer an agent who comforts a human patient and pushes an inanimate object to an agent who caresses an inanimate object and pushes a human patient. If their preferences were primarily based on the valence of the agent’s action directed towards the inanimate object, then one would expect them to prefer the agent who caressed the inanimate object over the agent who pushed it. This expectation is contradicted by the results of our experiment. If toddlers and infants’ preferences depended on the valence of the agent’s action irrespective of the target of this action, one would expect them to express no preference for either agent, which is not what we found in our experiments. Instead, what our data suggest is that toddlers and infants are already evaluating an agent based on how they treat another human. Thus, our results are consistent with the hypothesis that toddlers and infants’ preferences primarily reflect the valence of an agent’s action directed towards a human patient, not towards an inanimate object.

Our results could be due to the fact that for toddlers and infants, actions directed towards humans are more salient, more memorable, or receive a larger evaluative weight than actions directed towards inanimate objects. This interpretation is compatible with the claim by Premack and Premack [8] that there exists, in early human infancy, a core cognitive ability for the social evaluation of human agents based on their actions towards conspecifics. It is also compatible with several experiments showing that infants express early social preferences toward benevolent agents who performed positive actions toward their conspecifics [9]–[11], [15]. We extended these findings by providing evidence that infants are able to distinguish a benevolent from a malevolent agent whose action impacts a patient’s physical integrity.

Thus, in order to account for our results, we need to postulate a combination of at least two psychological components, one of which is sensitive to the nature of the patient and/or to the valence of her emotional states, and the other of which is sensitive to the nature of the agent’s action. Importantly, these two components have to be *combined* in a specific way in order to account for the results that we observed in our experiments.

We consider three nonexclusive theoretical possibilities for such combinations, ranging from the least to the most complex. A first simple possibility could be that infants are merely *associating* the identity of the agent of the action with the *emotional change* displayed by the patient, without really attending to the structure or valence of the agent’s action. That is, infants would notice that one character is always associated with the patient’s changing from happy to sad, and the other with the patient’s changing from sad to happy. Such an associative mechanism predicts that infants should prefer the latter to the former, even if the agent performs an action that is not harmful for the patient (i.e., dancing, spinning, etc.).

A second and more complex possibility could be that infants are associating an agent with the positive or negative valence of his or her action (i.e., throwing vs. raising), irrespective of the patient’s emotional response. Since, however, the association is stronger when the target of the agent’s action is a human patient than an inanimate object, the associative mechanism must further depend on some attentional mechanism geared towards the detection of human beings. Moreover, whether this associative mechanism could bypass the need for a causal analysis of the agent’s action is an open question.

A third possibility is that infants track not only the victim’s emotional response, but also the causal structure of the agent’s action, and that they assign blame for the victim’s suffering to the human agent who is causally responsible for the suffering (see [26], [27]). Although we know that infants are sensitive to the causal structure of events [28], [29], we do not know whether they can use this kind of analyses in social evaluation.

On the surface, the three aforementioned mechanisms are quite different in terms of the cognitive resources they require. Unfortunately, they cannot be fully disentangled on the basis of our present findings. Yet, other published results involving older populations suggest possible ways to address this issue. For instance, Leslie et al. [17] tested preschoolers in a situation where the omission of an act (refusing to give a cookie upon request) caused a patient to cry. Preschoolers did not evaluate the agent negatively, even though he was *associated* with the patient’s increased distress. This could be taken as evidence that, contrary to the first hypothesis, the nature of the action (i.e., causing physical harm versus omitting to give) matters, and not merely associations with an emotional change (as proposed by the first hypothesis). Vaish, Carpenter and Tomasello [30] presented 18-month-old toddlers with a human patient victimized by an agent through the destruction of his drawing. Despite the fact that the patient did not display an emotional reaction, toddlers were more likely to help the victimized patient than the non-victimized one. This could be taken as evidence that preschoolers are able to track the intrinsic valence of actions, irrespective of the patients’ emotional cues, as proposed by the second hypothesis. Buon et al. [31] showed that even when performing a concurrent linguistic task, adults evaluate more negatively an agent who performs an action (swinging) that causes harm to a victim (falling) than an agent who performs the same physical movements but does not cause the victim’s suffering (because the victim falls on her own). This suggests that, in adults, complex cognitive/linguistic resources are not necessary to perform blame assignment based on the causal structure of the event, as proposed by the third hypothesis.

These studies show that it is possible, in principle, to dissociate the three hypotheses, although the relevant experimental conditions must be adapted to preverbal infants. Meanwhile, it is important to note that even if 10-month-olds were basing their responses on simple associative mechanisms, the associations should link at least two factors, one of which is the structure of the *action*, and the other is the nature of the *patient* (or her emotional responses). Specifically, infants prefer agents who perform a positive action towards a human patient, and a negative one towards an inanimate object than agents who do the converse. Such a capacity would, in practice, enable infants to detect potentially harmful conspecifics, which, on evolutionary grounds, is useful to their survival. Before closing, we raise four questions for further research: the potential asymmetry between positive and negative actions, the small size of our effects, the role of intentions versus causal role and the link between the Help/Hinder situation and the Comfort/Harm situation.

### Positive versus Negative Actions

The first question is: to what extent do respectively the aversion prompted by an agent’s negative action and the appeal of an agent’s positive action play a symmetrical role in action-based social evaluations by preverbal human infants? Given the evolutionary importance of the detection of potentially harmful and dangerous agents, one would expect that the ability to detect harmful agents plays a stronger role and arises earlier than the ability to track benefactors. However, on the basis of our experimental design, which directly compares positive and negative actions, it is not possible to tease apart the respective contribution of the positive and negative outcomes of an agent’s act to infants’ evaluation of agents.

There is, however, some independent evidence suggesting that human infants give more weight to negative than to positive emotions. This “negativity bias” appears to emerge within the first year of life and has been documented through several research paradigms. For instance, Mumme and Fernald [32] found that 12-month-old infants display emotional contagion after watching television scenarios conveying negative emotions, but not positive ones. Vaish et al. [33]argue that in social referencing paradigms, negative cues (such as fear and disgust) have a more immediate and greater impact than positive cues. Cacioppo and Gardner [34] argue that negative information serves as a signal to change behavior whereas positive information is more likely to serve as a signal to stay on course. In this context, the negativity bias may serve the crucial evolutionary adaptive function of helping infants to avoid potentially harmful stimuli.

In our experiment, evidence for this hypothetical asymmetry comes from the toddlers’ results. When analyzed separately, toddlers’ absolute evaluations were significantly negative for the anti-social agent, but neither negative nor positive for the pro-social agent. In addition, toddlers made significantly more comments about negative than positive actions (if performed on humans). This suggests a greater saliency of anti-social actions than pro-social ones. Consistent with this, Hamlin, Wynn & Bloom [10] reported a looking time difference in 3-month-olds between the hinderer and a neutral agent, but not between a helper and a neutral agent. Note that this asymmetry between the processing of positive versus negative interactions was not found at six months [9], since infants were able to distinguish both the helper and the hinderer from the neutral control. This lack of asymmetry at 6-months could, however, be due to a ceiling effect, as in Hamlin et al.’s experimental situations [9], [10], the agents perform only one action (*either* positive *or* negative). In our experiment, each agent performs a pair of actions (one positive and one negative), thereby providing more opportunity for a potential asymmetry to show up. For instance, it could be that the positive action performed towards the human patient by the pro-social agent is partially counterbalanced by the negative action that the same agent performs on the object, which could also be negatively valued by young children and infants [35], yielding a relatively neutral evaluation of this agent. In contrast, the anti-social agent’s negative actions towards a human patient would not be counterbalanced by his positive actions towards objects, yielding a negative aggregate evaluation. More research is needed, using different neutral situations as baselines, to fully test a potential “negativity bias” and the conditions of its expression in the case of the social evaluation of agents based on their actions.

### Small Effect Size

Secondly, we have to acknowledge here that our effect size is small compared to those in some others experiments. For example, Hamlin and collaborators showed that infants prefer to interact with an agent who helps over an agent who hinders another to achieve his goal with only 16 participants (only two of them having failed to choose the helper on the test phase). Several hypotheses could account for this large difference in the sample size required. First, as we just noticed, the situations presented to infants and toddlers in our experiments are more complex than the situations presented to infants in Hamlin et al.’s experiment. In Hamlin et al.’s experiment, there are two agents and one patient, each of them performing only one action. In our experiment, there are two agents and two patients. In addition, each animated agent performed two sets of different actions and this could be more costly to process. Moreover, as mentioned above, we counterbalanced all emotional cues associated with both the agents and the victims to ensure that infants’ and toddlers’ responses were not only based on the emotional cues displayed by different actors. However, while this caution allowed us to explain the nature of our effects, it might also have weakened it. Indeed, while we would attribute a positive evaluation to a character who comforts a little girl, this positive evaluation could be dampened by the threatening cues associated with the action of “hitting a bag”.

### Intentions to Harm versus Harmful Consequences

The third question is that even though our study controls for low-level effects, it does incorporate a high-level confound: while the anti-social agent has an anti-social intention, he also causes actual harm to a human. Similarly, the pro-social agent has a pro-social intention and also provides comfort to a human. In adults, both intentions and consequences play a role in moral evaluation. However, in case a conflict arises, as in attempted harm, or in unintended harm, intentions are typically assigned a stronger weight than consequences [31], [36], [37]. Here, since intentions and consequences are confounded, we cannot tell whether infants and toddlers were more sensitive to one or the other. Other studies, however, have looked at the role of intentions in early social moral evaluations. For instance, Vaish and collaborators [15] found that preschoolers were more likely to help a neutral agent rather than a malevolent agent even if the latter agent’s action failed (attempted malevolent action). Hamlin [38] recently showed that 8-month-olds preferred a puppet who attempted to help a patient to reach its goal over a puppet who attempted to prevent it from achieving its goal, irrespective of whether the attempt succeeded or failed. However, 8-month-olds did not distinguish between two agents that had matched (helpful or harmful) intentions, and differed on the success or failure of their actions. In contrast to these studies suggesting the primacy of intentions over outcomes in infant’s social evaluations, others have showed that preschoolers are prone to use information about the consequences of an agent’s action rather than information about the agent’s intention [21], [39]. Finally, some researchers showed that preschoolers tend to use equally information about consequences and intentions [16], [40]. More research is needed to disentangle the respective roles of intentions and consequences as a function of experimental situations and paradigms during development.

### Help/Hinder versus Comfort/Harm Situations

The fourth question emerges from the fact that Premack and Premack [8] seemed to assume that the mechanism underlying social evaluation in preverbal human infants takes as input acts exemplifying either the Help/Hinder contrast or the Comfort/Harm contrast. As already mentioned, from a conceptual standpoint, there is room for drawing a distinction: an agent could not help or hinder someone else unless the latter had a goal; but an agent can harm or comfort a victim, whether or not the victim is pursuing a specific goal. Grasping the distinction between a joint action and an antagonistic action is necessary for understanding the contrast between helping and hindering, not the contrast between comforting and harming. What is required to grasp the contrast between harming and comforting is that the former, not the latter, causes the victim’s pain or distress.

It is therefore an open question whether a single cognitive mechanism underlies the responses to these two contrasts (help/hinder and comfort/harm), as hypothesized by Premack and Premack [8].

One possible speculation to be further investigated is that what underlies infants’ responses to instances of both the Help/Hinder and the Harm/Comfort contrasts (as well as other malevolent/benevolent contrasts) is a single mechanism for tracking the causation of *physical* and *psychological* distress. Whereas physical aggression causes the former, the frustration caused by either the failure to achieve a goal or the unequal distribution of resources could well induce the latter. It is reasonable to assume that young infants, because of their difficulties in carrying out planned actions, might have considerable experience with goal frustration, and might come to readily associate such situations with a state of psychological distress. Even though we are not aware of a direct test of this hypothesis, there is evidence that toddlers go to considerable trouble to help perfect strangers who are stuck in a situation where they cannot reach their goals [41]–[43]. If so, then it would follow that they will interpret the hinderer’s act in Hamlin et al.’s [9] study as causing the victim’s psychological distress (mutatis mutandis for help and psychological relief). An interesting research agenda, therefore, is to study whether the two situations studied by Premack and Premack [8] develop at the same pace in infants, and whether they involve a common mechanism whose function is to track the causation of physical or psychological pain.

## Supporting Information

Table S1 **Results obtained for each question asked in Experiment 1.** ^1^In order to know whether the mean obtained for each question is above chance level, we computed a one sample t-test against 0.^ 2^Only one actor is presented. ^3^the first and the second questionnaire are significantly correlated (r (28) = .567, p<.002). ^4^The two actors are presented side by side. n.s : non-significant.
(PDF)

Table S2 **Raw results of Experiment 2.**
(PDF)

Experiment S1 **Rating of agent’s expressions as a function of target type (animate vs. inanimate).**
(PDF)
