# Supplementary material for: Friend or Foe? Early Social Evaluation of Human Interactions
Source: PLoS One. 2014 Feb 19;9(2):e88612. doi: 10.1371/journal.pone.0088612 (PMC3929526; doi:10.1371/journal.pone.0088612)
Supplement: Table S1 — Results obtained for each question asked in Experiment 1. 1In order to know whether the mean obtained for each question is above chance level, we computed a one sample t-test against 0. 2Only one actor is presented. 3the first and the second questionnaire are significantly correlated (r (28) = .567, p<.002). 4The two actors are presented side by side. n.s : non-significant. (PDF) [file pone.0088612.s001.pdf]

Table S1: Results obtained for each question asked in Experiment 1

Questionnaires	Questions	Mean (SD)	Statistics[1]
First questionnaire[2]	Do you like him?	0.28 (0.16)	t(27) = 1.68 [n.s]
	Is he a good guy or a bad guy?	0.05 (0.16)	t(27) <0 [n.s]
	Is he nice looking or ugly?	0.0 (0.15)	t(27) <0 [n.s]
	Is he scary or nice?	-0.1 (0.15)	t(27) <0 [n.s]
	Do you want to play with him or not?	0.16 (0.18)	t(27) <0 [n.s]
	Differences between questions		F(4,17)<1 [n.s]
Second questionnaire[2,3]	Do you like him?	0.01 (0.18)	t(27) <0 [n.s]
	Is he a good guy or a bad guy?	0.07 (0.17)	t(27) <0 [n.s]
	Is he nice looking or ugly?	0.03 (0.14)	t(27) <0 [n.s]
	Is he scary or nice?	0.23 (0.14)	t(27) = 1.60 [n.s]
	Do you want to play with him or not?	-0.1 (0.18)	t(27) <0 [n.s]
	Difference between questions		F(4,17)<1 [n.s]
Third questionnaire[3]	Is he a good guy or a bad guy? (left)	0.19 (0.16)	t(27) = 1.17 [n.s]
	Is he a good guy or a bad guy? (right)	-0.5 (0.17)	t(27) <0 [n.s]
	Difference between questions left/right		F(1,20)<1[n.s]
	Which one is the nice guy?	0.18 (0.14)	t(27) = 1.23 [n.s]
	Which one is the bad guy?	0.14 (0.13)	t(27) = 1.07 [n.s]
	Which one would you like to play with?	0.29 (0.13)	t(27) = 2.12 [n.s]
	Difference between contrastive question[illegible]		F(2,19)<1[n.s]
