# Supplementary material for: Friend or Foe? Early Social Evaluation of Human Interactions
Source: PLoS One. 2014 Feb 19;9(2):e88612. doi: 10.1371/journal.pone.0088612 (PMC3929526; doi:10.1371/journal.pone.0088612)
Supplement: Table S2 — Raw results of Experiment 2. (PDF) [file pone.0088612.s002.pdf]

Table S2: Raw results of Experiment 2

				Coder 1				Coder 2			
subject	antisocial agent	first agent	first action	choice 1	choice 2	choice 3	choice 4	choice 1	choice 2	choice 3	choice 4
1	actor_A	actor_B	neg	1	0	0	0	1	0	0	0
2	actor_A	actor_A	neg	0	0	0	0	0	0	0	0
3	actor_B	actor_A	neg	1	-1	0	0	-1	1	0	0
4	actor_A	actor_B	pos	-1	1	-1	0	0	1	-1	0
5	actor_B	actor_B	pos	1	1	-1	1	1	1	-1	1
6	actor_B	actor_A	pos	1	-1	1	-1	1	-1	1	-1
7	actor_A	actor_A	pos	-1	-1	-1	0	-1	-1	-1	0
8	actor_B	actor_B	neg	1	1	-1	1	-1	1	-1	1
9	actor_A	actor_B	neg	1	1	1	-1	1	1	1	-1
10	actor_A	actor_B	neg	1	-1	1	0	-1	-1	1	1
11	actor_A	actor_A	neg	-1	1	-1	-1	-1	-1	-1	-1
12	actor_A	actor_B	pos	1	-1	1	1	1	-1	1	1
13	actor_B	actor_B	pos	1	1	0	0	-1	1	0	0
14	actor_B	actor_A	pos	1	1	-1	-1	1	0	0	0
15	actor_A	actor_A	pos	1	-1	1	-1	0	-1	1	0
16	actor_B	actor_B	neg	1	1	-1	1	1	1	-1	1
17	actor_A	actor_B	neg	1	-1	1	1	1	-1	1	1
18	actor_A	actor_A	neg	1	-1	1	-1	1	-1	1	-1
19	actor_B	actor_A	neg	1	1	-1	-1	1	0	0	0
20	actor_B	actor_A	neg	1	1	-1	0	1	1	-1	1
21	actor_A	actor_B	pos	-1	1	1	0	1	-1	-1	1
22	actor_B	actor_B	pos	-1	-1	1	-1	-1	-1	1	-1
23	actor_B	actor_A	pos	-1	-1	1	-1	1	-1	1	-1
24	actor_A	actor_A	pos	1	-1	1	-1	1	-1	1	-1
25	actor_B	actor_B	neg	1	1	-1	-1	1	1	-1	-1
26	actor_B	actor_B	neg	-1	1	-1	1	-1	1	-1	1
27	actor_A	actor_B	neg	1	1	1	-1	-1	1	1	-1
28	actor_A	actor_A	neg	1	1	0	0	1	-1	0	0
29	actor_B	actor_A	neg	1	-1	1	-1	1	-1	1	-1
30	actor_A	actor_B	pos	-1	-1	1	-1	-1	0	1	-1
31	actor_B	actor_B	pos	-1	1	1	-1	-1	1	1	-1
32	actor_B	actor_B	pos	1	-1	1	1	1	-1	1	1
33	actor_B	actor_A	pos	1	-1	1	0	1	-1	1	0
34	actor_A	actor_A	pos	-1	1	-1	1	-1	-1	-1	1
35	actor_B	actor_B	neg	1	1	1	-1	1	1	1	-1
36	actor_A	actor_B	neg	-1	1	-1	1	-1	1	-1	-1
37	actor_A	actor_A	neg	1	1	-1	1	1	1	-1	1
38	actor_B	actor_A	neg	0	1	-1	-1	1	1	-1	-1
39	actor_A	actor_B	pos	1	-1	-1	1	1	-1	1	1
40	actor_B	actor_B	pos	1	1	-1	1	1	1	-1	1
41	actor_B	actor_A	pos	1	1	1	1	1	1	1	-1
42	actor_A	actor_A	pos	1	-1	1	1	-1	-1	1	1
43	actor_B	actor_B	neg	-1	1	1	1	-1	1	1	1
44	actor_B	actor_A	neg	1	1	-1	1	-1	1	-1	1
45	actor_A	actor_A	pos	-1	0	-1	0	-1	1	-1	1
46	actor_B	actor_A	pos	1	1	1	0	1	1	1	0
47	actor_B	actor_A	pos	1	1	-1	1	1	1	-1	1
