# Supplementary material for: Friend or Foe? Early Social Evaluation of Human Interactions
Source: PLoS One. 2014 Feb 19;9(2):e88612. doi: 10.1371/journal.pone.0088612 (PMC3929526; doi:10.1371/journal.pone.0088612)
Supplement: Experiment S1 — Rating of agent’s expressions as a function of target type (animate vs. inanimate). (PDF) [file pone.0088612.s003.pdf]

Supplementary Experiment S1: Rating of agent's expressions as a function of target type (animate vs. inanimate)

Even though we asked the actors in the movie clips of Experiments 1 and 2 to express the same emotions towards the human patient (little girl) and the non-human patient (backpack), it is possible that they could not fully control their expressions as required. This could bias our results, especially if the positive/negative valence happened to be modulated as a function of the animacy of the target. For example, if the actors were more prone to express positive emotions towards the little girl than towards the backpack, the social preferences for the pro-social agent found in infants and toddlers could have been due, not to the pro-social nature of t their actions, but rather to the average intensity of their positive emotions.

In order to control for this possibility, we ran two additional experiments (on stimuli respectively used in Experiment 1 and in Experiment 2) on two groups of healthy adults who were asked to rate the emotional expressions of agents while the target of their actions (human being vs. inanimate object) was hidden by a static mask (figure S1).

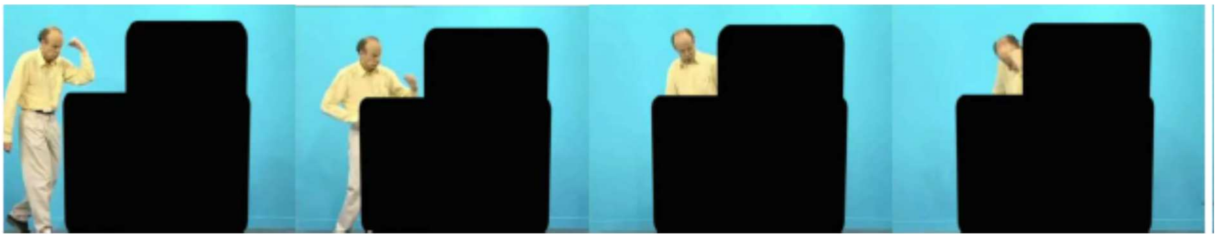

Figure S1. We displayed the movies used in our Experiment 1 and 2 with a visual mask enabling the viewer to see the facial expressions of the agents, but masking the human or non-human patients.

Procedure

Participants were recruited online via Amazon Mechanical Turk (https://www.mturk.com/mturk/). The instructions were given as follows: "*You will see a short video clip. After the clip, you will have to answer two simple questions about the actor in the clip. You can watch the clip only once and have to answer as quickly as possible! Please watch the video clip now by clicking below*. ».

Participants had then to watch one of the 8 video clips of the experiment; each video clip involved only one of the two actors (actor A vs. B), one of the two actions (hitting vs. comforting), and one of the two targets (human being vs. inanimate object). After having watched the video clip, participants had to answer the following question: "What does the actor express?" on a 7 –points scale running from (1): "an extremely positive emotion" to (7): "an extremely negative emotion".

Ratings for Experiment 1

Participants. Sixty-four participants were recruited via Amazon Mechanical Turk for this experiment. They had to perform the experiment on line and were paid 5 cents for their participation. Of the 64 participants, 10 were not analyzed because they responded too slowly (more than two standard deviations above the mean, more than 83 seconds, N=5), too fast (more than two standard deviations below the mean, less than 25 seconds, N=3), or because their rating deviated for more than 2 standard deviations from the mean response for a given sequence (N=2).

Results. We performed a general linear model analysis on the response given by the 54 remaining participants using the mean rating of emotional expressions as dependent measure and actor (A vs. B), action (positive vs. negative) and target of action (human patient vs. inanimate object) as independent factor. We found a main effect of action ($F(1,53)= 50.34$, $p<.0001$, $\eta p^2=.52$) indicating that overall emotional expressions of actors who comfort a target were evaluated more positively than emotional expressions of actors who hit a target. We also found a main effect of actor ($F(1,53)=19.25, p<.0001$, $\eta p^2=.29$) and an actor by target interaction ($F(1,53)=5.17, p<.05$, $\eta p^2=.10$) indicating that one of the actors was evaluated more positively than the other, but only when the target of the action was the human being (difference between the actors for the human target: $F(1,53)=5.17$, $p<.05$, $\eta p^2=.28$), for the inanimate target: ($F(1,53) =1.32$, $p>.1$, $\eta p^2=.04$). In addition, we found an action by target interaction ($F(1,53) =5.12$, $p<.05$, $\eta p^2=.10$) indicating that participants rated the emotion of the actor more **positively** when his action was performed toward the **inanimate object** but only when actors performed the **positive action** (difference as a function of the target, action of hitting : $F(1,26)<1, p>.1$, action of caressing : $F(1,26)=7.51$, $p<0.1$, $\eta p^2=.23$).

Ratings for Experiment 2

Participants. Sixty-four participants took part in this study. Sixteen participants were removed from the analysis due to either too long (n=1) or too short (n=10) response times, or to failure to answer the experimental question (n=5). The remaining 48 participants were analyzed using a GLM with actor, target and action in the analysis.

Results We found no effect of actor ($F(1,47)=3.32$, $p>.1$, $\eta p^2=.03$), nor of target ($F(1,47)=1.73$, $p>.1$, $\mu^2=.01$), but a main effect of action with agents performing positive actions being evaluated more positively than for negative actions ($F(1,47)=69.10$, $p<.0001$, $\eta p^2=.42$) and an actor by action interaction ($F(1,47)=10.85$, $p<.01$, $\eta p^2=.021$). This interaction was due to the fact that the emotional expression of one actor was evaluated more positively than that of the other actor, but only when the action performed was positive ($F(1,23)=15.87$, $p<.0001$, $\eta p^2=.47$).

Discussion

In both experiments, we found that the actors were not completely equivalent in their rendering of their emotions across the movies. In Experiment 1, overall one actor had more positive affects than the other, and especially when interacting with a human target. In Experiment 2, the two actors were equivalent in their overall expressions, but one had more positive affect when performing positive action than the other. These differences, which are inevitable when using real actors as opposed to animated cartoons or puppets, could not have influenced our results, since the actors, the actions and their targets were globally counterbalanced across participants, and hence these effects average out across participants without affecting our main outcome (which is the action by target interaction).

The only potentially damaging effect would be to have an action by target interaction. Such an interaction was found in Experiment 1. However, it was found to be in the opposite direction of the observed effects. Indeed, for some reasons, it was somewhat easier/more natural for the actors to express positive facial and bodily expressions while comforting the inanimate object than while comforting a little girl. This should have produced an overall more positive assessment of the anti-social agent than the pro-social agent, which is the opposite of what we found. In Experiment 2, no such interaction was found.
